# Phenotypic plasticity in sex pheromone production in *Bicyclus anynana* butterflies

**DOI:** 10.1038/srep39002

**Published:** 2016-12-14

**Authors:** Emilie Dion, Antónia Monteiro, Joanne Y. Yew

**Affiliations:** 1 Department of Biological Sciences, 14 Science Drive 4, National University of Singapore, 117543, Singapore; 2 Temasek Life Sciences Laboratory, 1 Research Link, 118173, Singapore; 3 Yale-NUS College, 6 College Avenue East, 138614, Singapore; 4 Pacific Biosciences Research Center, 1993 East West Road, University of Hawai’i at Mānoa, Honolulu, HI 96822, USA

## Abstract

Phenotypic plasticity refers to the environmental control of phenotypes. Cues experienced during development (developmental plasticity) or during adulthood (acclimatization) can both affect adult phenotypes. Phenotypic plasticity has been described in many traits but examples of developmental plasticity in physiological traits, in particular, remain scarce. We examined developmental plasticity and acclimatization in pheromone production in the butterfly *Bicyclus anynana* in response to rearing temperature. *B. anynana* lives in the African tropics where warm rearing temperatures of the wet season produce active males that court and females that choose, whereas cooler temperatures of the dry season lead to choosy less active males and courting females. We hypothesized that if male pheromone production is costly, it should be reduced in the dry season form. After describing the ultrastructure of pheromone producing cells, we showed that dry season males produced significantly less sex pheromones than wet season males, partly due to acclimatization and partly due to developmental plasticity. Variation in levels of one of the compounds is associated with differential regulation of a pheromone biosynthetic enzyme gene. This plasticity might be an adaptation to minimize pheromone production costs during the stressful dry season.

Phenotypic plasticity is the ability of a genotype to produce different phenotypes in response to environmental cues such as diet, photoperiod, or temperature^1^,^2^. This phenomenon can be divided into two main components: (a) phenotypic flexibility, or acclimatization, whereby phenotypes undergo reversible changes during adulthood upon exposure to different environments, and (b) developmental plasticity, where environmental conditions experienced during development lead to alternative adult phenotypes^2^,^3^. Typical cases of developmental plasticity include caste determination in social insects, such as termites or bees^4^,^5^, horn development in beetles^6^, wing polyphenism in locusts^7^, and wing colours and colour patterns in butterflies^8^,^9^,^10^,^11^. Developmental plasticity can be an adaptation to recurrent and predictable fluctuating environments, such as seasons, and is commonly found in organisms that have multiple generations in a year, as it is the case with most insects^12^. Environmental cues experienced during development are often predictive of adult environments, and some species have evolved mechanisms that sense these cues and steer the individual’s development into alternative phenotypes adapted to the particular expected adult environment^13^.

Developmental plasticity occurs in the form of a seasonal polyphenism for some butterfly species such as *Bicyclus anynana*, where two or more distinct phenotypes, without intermediates, are produced in response to environmental cues experienced during development. *B. anynana* is a subtropical African species which encounters, in its natural environment, the alternation of a wet, favorable warm season and a dry, cool, stressful season when food availability is reduced. Temperatures experienced during larval and pupal stages lead to the development of two distinct adult phenotypes adapted to the two main seasons: the wet season (WS) form and the dry season (DS) form^14^, which differ in morphology, behavior, and physiology. For example, WS individuals exhibit large eyespots and bright colours on the ventral sides of their wings, contrary to the duller patterns of the cryptic DS form^14^,^15^. In addition, WS males approach “choosy” WS females and engage in vigorous courtship, whereas DS males become the choosier sex while DS females do much of the courting^16^,^17^. The choosy behavior of males and females is focused on the sexual ornaments present on the dorsal forewings, the bright white, UV-reflective eyespot centers^18^. In WS males, in particular, these eyespot centers are larger and conspicuous, compared to the duller and smaller ones of the DS male^16^. These ornaments vary in size and brightness, and are correlated with the sex role reversal^16^,^19^. *B. anynana* females also display developmental plasticity for opsin receptor expression levels that reflect their variation in choosiness towards the visual ornaments in the wings of males. Females express all three opsin genes at lower levels in the DS form, when they become less choosy towards male wing color ornamentation^19^. In addition, adults exhibit phenotypic flexibility as well, whereby overall activity and metabolism varies according to ambient temperature. In warm conditions, when food availability is higher, both seasonal forms use energetic reserves at a higher rate, and become more active. For instance, they court more, leading to faster mating^20^.

In addition to visual signals, such as the eyespot color patterns described above, insects also use chemical signals, namely sex pheromones, to identify and choose potential mates^21^,^22^. Mating signals are thought to honestly advertise the identity and quality of the sender, and are expected to be costly to produce and maintain^21^,^23^. For instance, pheromone production in male *Drosophila grimshawi* is condition dependent, and leads to decreased longevity once it is produced^24^,^25^. Costs of pheromone production can also be indirect, such as when male sex pheromone attracts other competing males, predators or parasitoids^21^.

*B. anynana* males of the WS form release sex pheromones (named Male Sex Pheromones - MSPs) from a wing area containing modified scales called androconia^26^. The pheromone blend of *B. anynana* contains 3 lipid components, (*Z*)-9-tetradecenol, 1-hexadecanal and 6, 10, 14-trimethylpentadecan-2-ol (respectively termed MSP1, MSP2 and MSP3), which are employed at short range during the courtship sequence to influence female mate choice^26^,^27^. Costs of pheromone production have not yet been investigated in *B. anynana*, but according to Nieberding *et al*.^28^, the blend composition is an honest signal of male identity. In particular, MSP2 titers and its relative ratio to other MSP components are indicators of male age and inbreeding status^28^,^29^.

Given the presence of developmental plasticity in mate choosiness, and sexual ornament size and brightness in *B. anynana*, we asked whether this species also evolved a temperature-sensitive developmental mechanism that allows the reduction of predicted costs of pheromone production in cooler dry season conditions, when food availability is sparse, and when females perform a substantial amount of courtship. To explore this hypothesis, we first described the ultrastructure of the pheromone producing cells, found under the androconial scales on both wings of *B. anynana* males, and then quantified MSP titers in both male seasonal forms. Next, we analyzed the effect of developmental and adult rearing temperatures on blend component amounts. Lastly, we examined possible mechanisms of plasticity by quantifying expression levels of pheromone biosynthetic enzymes in both seasonal forms.

## Results

### The MSP-producing pheromone glands are located in the wings below the androconia

**Figure 1:**
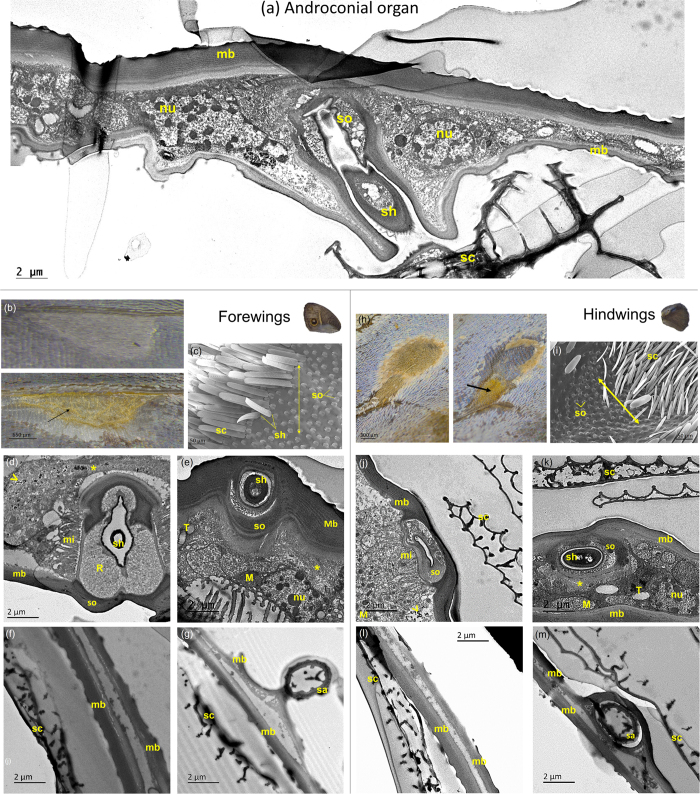
Light and transmission electron microscope (TEM) micrographs of forewing and hindwing androconia and underlying glands. (**a**) Cross section of the hindwing androconial organ of a DS-17 male. (**b**,**h**) show the forewing and the hindwing regions covered by modified scales, the androconia, (**b**, top image; **h**, left image) and with all scales removed on both sides of the wing (**b**, bottom; **h**, right). The two black arrows point to two yellow patches underneath the cuticle, which are likely to contain pheromone secretory cells. TEM cross-sections were done in these yellow areas along the axis indicated by the double arrow on SEM images (**c**,**i**) (scales were manually removed for the purpose of showing clearly the sockets at the surface). (**d**–**g**) Cross-sections done in the forewing; at the androconial organ, (**d**,**e**) and at a control area (**f**,**g**). Images (**j**) to (**m**) were taken from cross sections done in the hindwing, at the androconial organ (**j**,**k**) and at a control area (**l**,**m**). Images (**d**,**f**,**j**,**l**) were taken from WS-27 males whereas (**a**,**e**,**g**,**k**,**m**) are from DS-17 males. SEM and TEM images display scales (sc) with shafts (sh) that penetrate into socket cells, the wing membrane (mb), and the fine structures between the two wing membranes. The cellular components include: nucleus (nu), microvilli (mi), mitochondria (M), reservoir (R), tracheas (T), endoplasmic reticulum (*), and glycogen droplets shown by arrowheads. More pictures, at lower magnification, are available in Supplementary Figure 2.

The forewings and hindwings of *B. anynana* contain secretory cells below the androconia where the MSPs are likely to be produced. Upon removal of the androconial scales from the surface of the forewing, a thick yellow area appeared underneath, crossed by many micro-veins (Fig. 1b and c). On the hindwing, the yellow area was situated under a triangular patch of long thin androconial scales close to a main vein (Fig. 1h). Scales are maintained at the surface by their shafts, inserted into the socket cells (Fig. 1a and c). Transmission electron microscopy (TEM) of cross-sections in the yellow areas revealed live cellular material contained between the dorsal and ventral wing membranes (Fig. 1a,d,e,j,k and Supplementary Fig. 2). The cells exhibit a large number of electron-dense granules, along with numerous clear vesicles likely containing MSP secretions that are probably discharged into a reservoir space surrounding the proximal tip of the scale, and immediately exterior to the socket cell (Fig. 1d and Supplementary Fig. 2). The socket cells in this region display a series of membrane protrusions or microvilli (Fig. 1d and j; Supplementary Fig. 2). Mitochondria are numerous in the socket cells (Fig. 1e,j,k; Supplementary Fig. 2), and the smooth-surfaced endoplasmic reticulum is also well developed (Fig. 1d,e,k, and Supplementary Fig. 2). These cellular components are common features of pheromone-producing glands^30^,^31^ and are found in both DS and WS males. Cross-sections made at the control wing areas display only a dorsal and ventral cuticular membrane, with a narrow and empty inter-membrane space, when viewed under TEM. Cellular components are missing in socket cells (Fig. 1f,g,l and m). Based on these observations, the pheromones are most likely synthesized in the cells below the androconia, rather than transported from a remote site and stored there.

### *B. anynana* display phenotypic plasticity in MSP levels on both forewings and hindwings

**Figure 2:**
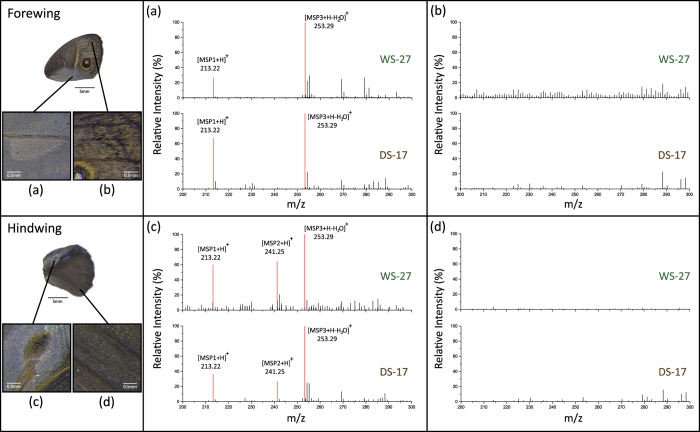
WS-27 and DS-17 males produce the same sex pheromone blend on their forewings and hindwings. Representative DART MS spectra of androconia sampled from the forewing (**a**), the hindwing (**c**); and from rubbed scales from the edge of the wing (as control, forewing (**b**) and hindwing (**d**)). The monoisotopic peak corresponding to each MSP is shown in red. Relative intensity is the height of a peak relative to the height of the most intense peak (defined as 100%) in the spectrum. The full spectra with m/z ranging from 200 to 700 are shown in Supplementary Figure 3.

To measure pheromone production directly from the androconia, we used Direct Analysis in Real Time mass spectrometry (DART-MS)^32^, which allows spatially-resolved chemical analysis directly from tissue, without extraction or purification^33^. Examination of androconia exudate or dissected androconia revealed that MSP secretion was localized to the androconia and that WS males maintained at high temperature during adulthood (WS-27) and DS males maintained at low temperature during adulthood (DS-17) have identical MSP blend compositions. MSP1 and MSP3 were detected from the androconia on the forewings, and the three MSPs were detected from the androconia on the hindwings in both seasonal forms (Fig. 2a). The two most abundant compounds on the forewing and hindwing were MSP1 and MSP3, with respective observed mass to charge (*m*/*z*) of 213.22 and 253.29. These values match the calculated [M + H]^+^ for MSP1 and [M + H − H_2_O]^+^ for MSP3 (Fig. 2a). MSP2 is not detected on forewings, but the ion with *m*/*z* 241.25 was found on the hindwing androconia of both seasonal forms, matching the calculated mass [M + H]^+^ of MSP2 (Fig. 2c). The control wing areas had no detectable signal corresponding to any of the MSPs (Fig. 2b and d).

**Figure 3:**
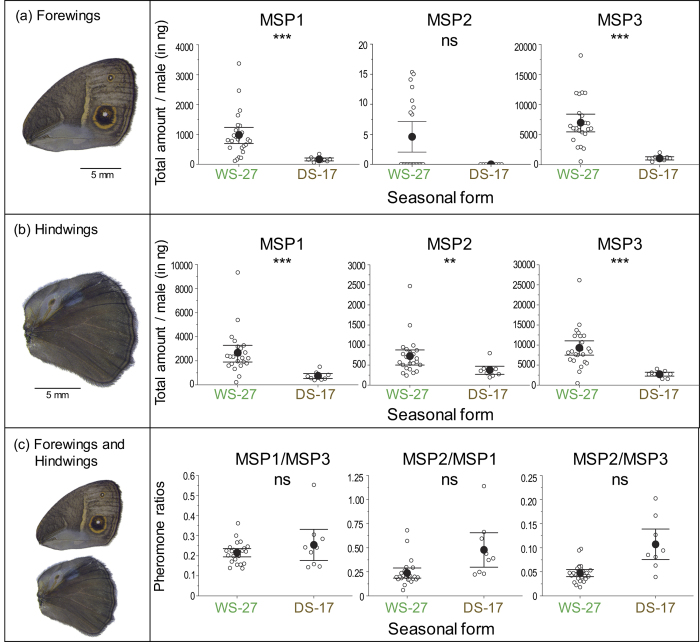
*B. anynana* is phenotypically plastic for MSP levels on both hindwings and forewings. Average MSP amount measured on the two forewings (**a**), the two hindwings (**b**) and ratios calculated from abundances of the 4 wings (forewing and hindwing values for the same animal were added) (**c**). Amounts were calculated from GCMS analysis. Vertical bars represent the 95% confidence intervals and the open circles are each data point measured. n(DS-17) = 10 males and n(WS-27) = 24 males. Asterisks (*) indicate *P* value, with ***P < 0.001, **P < 0.01, and ns = not significant.

Quantitative measurements of MSP levels were performed using gas chromatography MS (GCMS) on whole wing extracts of both seasonal forms (Supplementary Fig. 4). The position of the double bond in MSP1 was detected between carbon 9 and 10 (Δ9) in both WS-27 and DS-17 males (Supplementary Fig. 5). In general, DS-17 males produce significantly lower amounts of MSPs on both wings relative to WS-27 males (Fig. 3). On forewings, DS-17 males produce lower levels of MSP1 and MSP3 than WS-27 males, and males of both seasonal forms produce similarly low to undetectable amounts of MSP2 (Fig. 3a). The low intensity MSP2 signal is likely to be contamination from contact with the androconial hairpencils on the hindwings. On hindwings, DS-17 males produced significantly lower amounts of MSP1, MSP2, and MSP3 relative to WS-27 males (Fig. 3b). The relative ratios of the MSPs to each other (MSP1/MSP3, MSP2/MSP1 and MSP2/MSP3) from WS-27 and DS-17 males are similar (Fig. 3c). Statistical analyses are detailed in Table 1.

### MSP production is controlled by temperature experienced during development and adulthood

**Figure 4:**
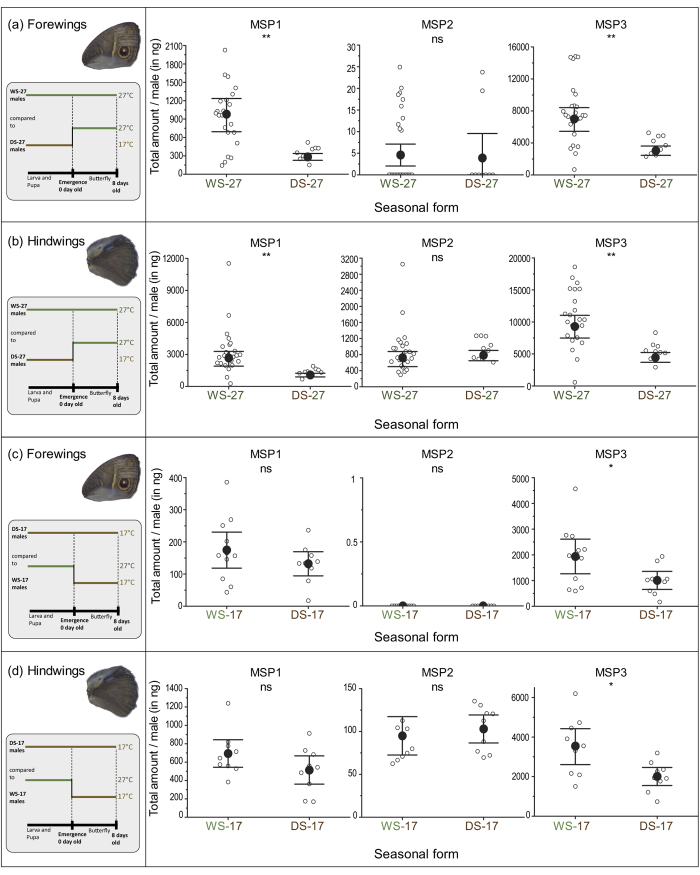
Plasticity of MSP levels is environmentally and developmentally controlled on both forewings and hindwings. (**a**) Average MSP amount measured on the two forewings and the 2 hindwings (**b**) of WS-27 and DS-27 males. (**c**) Average MSP amount measured on the two forewings and the 2 hindwings (**d**) of DS-17 and WS-17 males. Amounts were calculated from GCMS analyses. Vertical bars are 95% confidence intervals and open circles are each data point. n(WS-27) = 24, n(DS-27), n(DS-17) and n(WS-17) = 10 males each. Asterisks (*) indicate *P* value, with **P < 0.01,*P < 0.05, and ns = not significant.

**Figure 5:**
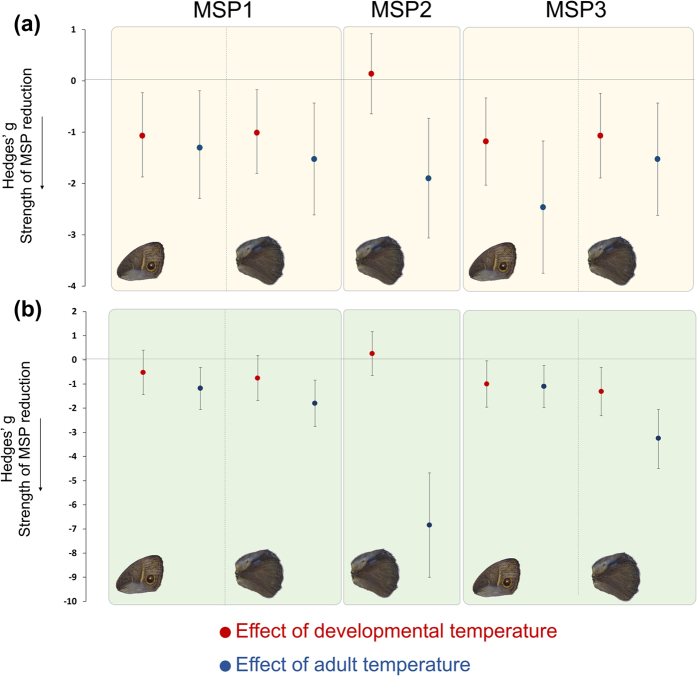
Adult and developmental environments impact MSP1 and MSP3 masses with similar strength. Hedges’ g effect sizes of pheromone levels due to adult temperatures (in blue, calculated from DS-17 and DS-27 in (**a**) and from WS-27 and WS-17 in (**b**)) and developmental temperatures (in red, calculated from DS-27 and WS-27 in (**a**) and from WS-17 and DS-17 in (**b**)) are equivalent for MSP1 and MSP3, as shown by overlapping 95% confidence intervals (error bars).

To test whether temperatures experienced during development alone could influence levels of pheromone production, we compared pheromone levels in WS and DS adult males placed immediately upon emergence at a common temperature of either 27 °C or 17 °C for 8 days. We first tested a common temperature of 27 °C (WS-27 and DS-27). Developmental temperature influenced levels of two of the MSPs; DS-27 males produced less MSP1 and MSP3 on both wings relative to WS-27 males, but produced equivalent levels of MSP2 on hindwings (Figs 4a,b and 5a and Table 1). This result indicates that the production of MSP1 and MSP3 is, in part, determined by the temperature experienced during development, whereas production of MSP2 is primarily dependent on the temperature experienced during adulthood. We repeated the same experiment with WS and DS reared butterflies but instead placed adults at 17 °C for 8 days immediately upon emergence (WS-17 and DS-17). DS-17 males produced less MSP3 on both wings relative to WS-17 males. DS-17 and WS-17 individuals, however, produced similar amounts of MSP1 and MSP2 (Figs 4c,d and 5b, and Table 1). All together, these results indicate that MSP1 and MSP3 levels are subject to developmental plasticity but MSP2 production is not.

To compare the relative contribution of developmental temperature and adult temperature on the amounts of each pheromone, we calculated Hedges’ g, which measures effect size. We found that temperature experienced during development and temperature experienced during adulthood produced similar changes in the amount of MSP1 and MSP3 on both wings, as shown by comparable magnitudes in their effect sizes. However, a change in MSP2 abundance on hindwings is influenced only by adult temperature, as the effect size due to developmental temperature is zero for this compound (Fig. 5).

### The *fatty acyl Δ11-desaturase* gene is differentially regulated in WS and DS males

**Figure 6:**
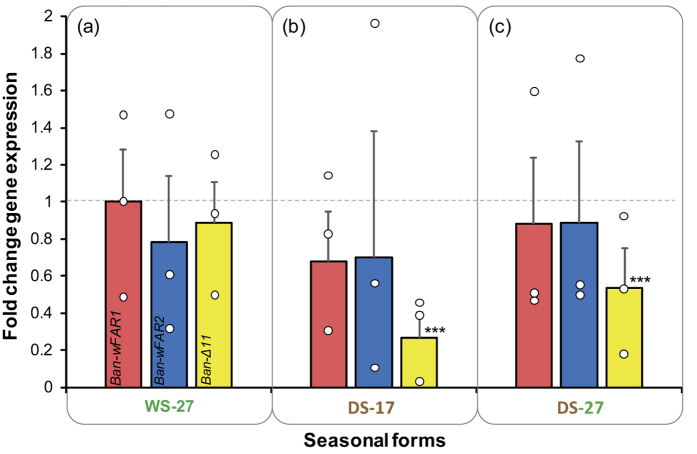
Expression of MSP1 and MSP2 producing enzymes extracted from the androconial organ on the forewing and the hindwing of males under different rearing temperature regimes. Bars show fold change expression relative to the baseline group (WS-27, value of 1, dotted line) using *EF1α* as an endogenous control. Bars represent mean ± SEM of 3 biological replicates, each consisting of a pool of 40 androconia dissected from 10 males. The red, blue and yellow colors correspond respectively to *Ban-wFAR1*, *Ban-wFAR2* and *Ban-Δ11* expression. Open circles are data points, and asterisks (***) indicate a significant difference from the gene expression in the baseline group at p < 0.01.

To identify molecular markers that could underlie the temperature-dependent changes in pheromone production, we quantified expression levels of 3 genes implicated in the synthesis of *B. anynana* MSPs. *Fatty acyl-reductase1* (*Ban-wFAR1*) is involved in the biosynthesis of MSP2, and *fatty acyl-reductase 2* (*Ban-wFAR2*) and *fatty acyl-desaturase Δ11* (*Ban-Δ11*) are both part of the biosynthesis of MSP1^34^. Androconial glands were sampled from three treatment groups: WS-27, DS-17 and DS-27 butterflies. *B. anynana* showed phenotypic plasticity in expression of *Ban-Δ11* but not in the other two genes (Randomisation tests; p < 0.001) (Fig. 6a and b). To test whether this plasticity was partly determined via temperatures experienced during development, we compared gene expression levels between WS-27 and DS-27 individuals (Fig. 6a and c). *Ban-Δ11* was expressed at lower levels in DS-27 as compared to WS-27 males (Randomisation tests; p < 0.001). The two fatty acyl-reductases in the two DS treatments (DS-17 and DS-27) were not differentially expressed relative to those of the WS-27 treatment (Randomisation tests – DS-27 vs WS-27: p = 0.710 for *Ban-wFAR1* and p = 0.810 for *Ban-wFAR2*; DS-17 vs WS-27: p = 0.610 for *Ban-wFAR1* and p = 0.720 for *Ban-wFAR2*) (Fig. 6).

## Discussion

Males produce less MSP1 and MSP3 when cooler developmental temperatures indicate the arrival of the dry season, and part of this response is not reversible by exposure to warmer adult temperatures. In contrast, MSP2 production appears to be primarily influenced by temperatures experienced during adulthood. Therefore, *B. anynana* exhibits temperature-induced developmental plasticity in the production of male sex pheromones, which is further regulated during adulthood by acclimatization. Taken together, the MSP blend, characterized by both absolute quantities and ratios of pheromones, is impacted by the butterfly’s developmental and adult temperatures. While developmental plasticity in pheromone production has previously been shown in honey bees^35^, the control of pheromone production genes by developmental temperatures is described here for the first time.

The developmental plasticity and acclimatization in MSP levels are likely mediated by different molecular and physiological mechanisms. The environmental control of pheromone production during adulthood can probably be ascribed to temperature dependencies of the enzymes involved in synthetizing the pheromones. As the temperature of a system decreases, the rate of enzymatic reactions will usually decrease as well^36^. The developmental control of plasticity, however, requires a different explanation. Developmental plasticity in MSP1 production is correlated with the lower *Ban-Δ11* expression in androconial glands of DS males compared to WS males. In *B. anynana* butterflies, the developmental control of a single step in the biosynthesis of MSP1 appears to be enough to significantly modify the amount of MSP1 produced. The fatty acyl Δ11-desaturase catalyses the formation of (*Z*)-11-hexadecenoic acid from the precursor palmitic acid, and constitutes the first step of MSP1 biosynthesis. Another enzyme, further down in the pathway, Ban-wFAR2, causes the reduction of (*Z*)-9-tetradecenoic acid towards (*Z*)-9-tetradecenol in the last step of its biosynthesis^34^. Since *Ban-wFAR2* mRNA levels were not affected by low developmental temperatures, the difference in the amount of MSP1 between WS and DS forms is likely associated with the different levels of expression of the enzyme controlling the first step of the biosynthesis pathway. The temperature-mediated mechanisms of *Ban*-Δ11 expression may involve the moulting hormone 20-Hydroxyecdysone (20E) and its receptor EcR. Rearing temperature was previously shown to alter levels of 20E in the hemolymph of late 5^th^ instar larvae, and these titre differences mediated plasticity in eyespot size and brightness of *B. anynana*^11^. In addition, 20E is known to regulate pheromone biosynthesis in flies and beetles, by increasing or reducing the synthesis of enzymes at the transcriptional level^37^,^38^,^39^. Unfortunately, the literature remains particularly poor regarding regulation of sex pheromone synthesis in butterflies.

Our results do not address the mechanism behind MSP3 developmental regulation. MSP3 is a phytol-derived compound that originates from chlorophyll-a degradation^40^,^41^. Similar to MSP1, the reduction in MSP3 production in DS males could be controlled by differential regulation of its biosynthetic enzymes. However, the complete biosynthetic process of MSP3 from phytol is not known in *B. anynana*.

We considered several alternative mechanisms for the observed developmental plasticity in pheromone production but later dismissed them. First, we hypothesized that number or size of gland cells could have been altered by developmental temperature. However, two observations led us to dismiss this hypothesis: i) expression levels of *Ban-wFAR1* and *Ban-wFAR2* in WS-27 and DS-17 males were equivalent and ii) androconia were of similar sizes in both seasonal forms (Supplementary Fig. 6). Second, we hypothesized that developmental temperature could have altered the timing at which particular blend components are first produced. However, we dismissed this hypothesis because none of the MSPs were detected by GCMS analysis on the day of eclosion^26^, but all were found by DART-MS from the wings of 2 day-old males of both seasonal forms (data not shown), indicating a coordinated onset of pheromone production. Moreover, quantification by GCMS revealed that at 3 days old, significant differences in MSP amounts were already evident between both seasonal forms (Supplementary Fig. 7). We cannot exclude the possibility, however, that developmental temperature alters the rate of enzymatic activity during adulthood. In this case, both a decrease in gene expression and a change in reaction rate would contribute to the difference in pheromone production.

The composition of the sex pheromone blend often provides reliable information about mate identity and quality, thus, changes in sex pheromone signals in a species are unexpected as they can disrupt mate recognition^21^,^22^,^27^. Despite these considerations, phenotypic plasticity in the sex pheromone profile has been documented in several species responding to different biotic and abiotic environments. In a predatory wasp, for instance, warmer larval rearing conditions induced a higher production of the scent marking secretions which attracted females receptive for copulation^42^. In *D. melanogaster*, component titres and ratios of male cuticular hydrocarbons were changed with adult social environment^43^,^44^. The sex pheromone blend also varies with nutrition in the parasitoid wasp *Nasonia vitripennis*^45^, and with mating status and amount of sugar intake in female tobacco budworms^46^. In most of these systems, however, it is still unclear whether the plasticity is adaptive, and how this plasticity is regulated.

Our results suggest that the temperature-mediated regulation of pheromone expression in males may be an evolved adaptation. DS males may have lower pheromone production abilities when developing at low temperature because of energetic trade-offs. During the dry season, food availability is scarce and butterflies experience long periods of starvation, when they remain quiescent and keep a low metabolism^14^,^20^. Because DS butterflies do not reproduce until the end of the dry season^47^,^48^, they probably invest their limited energetic resources into the development of traits that are most important for their survival and future reproduction. These traits may include immunocompetence^49^, olfaction (for food foraging for instance^50^,^51^), vision (for wing pattern discrimination^19^,^52^), or spermatophore quality^16^ (for reproductive investment). Moreover, high investment in pheromone production may not be as important for DS males as it is for WS males since DS females are less choosy, they perform higher amounts of courtship, and eagerly mate with most males. This eagerness to mate is likely due to females receiving a high quality spermatophore from DS males (but not from WS males) that allows them to live longer and lay more eggs^16^. Sex pheromones in DS males, thus, are less critical for attracting and convincing females to mate.

The acclimatization of adult DS pheromone levels to higher temperature may also be an evolved adaptation. Towards the end of the dry season, DS butterflies experience an increase in temperature, which induces a switch from a low metabolism and an inactive lifestyle, to a higher use of body fat and a more active behaviour, such as courting and mating^47^,^48^. The increase in sex pheromone amounts associated with higher ambient temperature is then likely to increase their chances of reproducing. Given that MSP2 amounts are not developmentally regulated, DS adults experiencing higher temperature should have higher MSP2 ratios than WS-27 males. High ratios of MSP2 relative to the two other MSPs have been shown to be more attractive to *B. anynana* females^28^. The partial developmental control of two of the three MSP and the acclimatization of all the MSPs might therefore be an adaptation. This system favours energy conservation and survivorship during most of the dry season, when female-solicited mating may primarily be taking place, however, when increasing temperatures finally cue the end of the dry season, male butterflies are able to rapidly acclimatize and produce more attractive MSP blends due to the elevated MSP production with different component ratios. This increased investment by DS males in metabolically expensive sex pheromones may be explained by *B. anynana* exhibiting last-male sperm precedence^53^,^54^, leading to increased chances of paternity for the most attractive males, when females start reproducing at the end of the dry season.

The ultrastructure examination of the androconial organ showed that males from both seasonal forms exhibit live gland cells on their wings. On the forewings, the gland is located in the area delimited by the androconia, whereas on the hindwings, the gland is located in the yellow triangular swollen area underneath thin long androconial scales. The drop-shaped area nearby is filled with wide and large dark-coloured scales (they appear lighter in Fig. 1 due to over-exposure), placed in a concave surface on the wing. Given their proximity to the gland, we hypothesize that these dark-coloured scales may absorb more light, leading to an increase in temperature that, in turn, may help the vaporization of the pheromones. Wing androconia have been thoroughly described in several moth species^55^, as well as in Milkweed^56^ or *Danaini*^57^ butterflies; however, the ultrastructure of the male wing androconial organ that lies underneath these scales is, to our knowledge, described here for the first time. Cytological features of the androconial organ from the two *B. anynana* seasonal forms are typical of insect exocrine glands and commonly found in sex pheromone-producing cells in other Lepidopteran^30^,^31^,^58^. The cells contain many mitochondria, lipid droplets, endoplasmic reticulum, and apical membrane protrusions (microvilli) which increase the surface of release into what is a probable reservoir – an extra cellular space enabling the storage of secretions^59^ - surrounding the scale socket. Pheromones might be released into the external environment from the cavity of the socket cell, where it contacts the base of the scale cell.

In conclusion, examples of developmental plasticity in traits under sexual selection remain rare, especially in sex pheromone emission, because changes in the blend could be perceived as less attractive, and thus be selected against^60^. However, the results presented in this study demonstrate that male sex pheromone production is controlled by temperature experienced during development and during adulthood in *B. anynana*. Changes in levels of some MSP compounds correlate with the differential regulation of genes coding for enzymes involved in MSP synthesis. Sex pheromone developmental plasticity and flexibility may be an adaptation to the seasonal environment, potentially allowing the sex-role reversed DS choosy males to reduce predicted costs of pheromone production to help them survive the stressful dry season, while still allowing them to produce an attractive pheromone blend to help them gain paternity when rising temperatures cue the arrival of the wet season.

## Methods

### Insect rearing

*B. anynana* larvae were reared on young corn plants at 17 °C or 27 °C in 2 different climate rooms, both with a 12:12 h light:dark cycle and 70% relative humidity. Animals were sexed at the pupal stage and separated into plastic containers. Every morning, upon emergence, adults were transferred to individual cages and fed mashed banana ad libitum. Individuals reared at 27 °C yielded wet season forms and those reared at 17 °C produced dry season forms (referred respectively as WS and DS). To assess developmental plasticity in pheromone production, some butterflies were shifted from one temperature to the other on the day of emergence. We used the following notation: WS-27 and DS-17 males were kept throughout their lives at 27 °C and 17 °C, respectively; DS-27 males were reared as larvae and pupae at 17 °C and transferred to 27 °C upon emergence; WS-17 males were reared as larvae and pupae at 27 °C and then transferred to 17 °C upon emergence (Supplementary Fig. 1). All butterflies used in this study were 8 days old, of equivalent sizes and with wings in good condition. Treatments were conducted in individuals across multiple generations.

### Transmission and Scanning Electron Microscopy (TEM and SEM)

Androconial organ samples for TEM were fixed overnight in a solution of 2.5% glutaraldehyde in 0.1 M phosphate buffer (pH 7.2) at 4 °C, washed three times in 0.1 M phosphate buffer, and additionally fixed in 1% osmium tetroxide in 0.1 M phosphate buffer for 1 hour at 4 °C. The samples were gradually dehydrated with ethanol, embedded in Spurr’s resin, and sectioned on an ultramicrotome (Lecia Ultracut UCT). Ultrathin sections were double stained with uranyl acetate and lead citrate and analyzed with a JEOL JEM-1230 electron microscope at 120 kV. Three DS and three WS males were used. For SEM, samples were mounted on aluminium stubs and sputter-coated with a thin layer of platinum using a JEOL JFC 1100 ion sputter. The samples were analyzed with a JEOL JSM 6510 SEM. Light microscopy images were obtained with a Leica MMS1000 microscope and the associated software (Leica Application Suite v.4.4.0, 2013).

### Direct Analysis in Real Time (DART)-MS

Direct analysis of wings was performed with an atmospheric pressure ionization time-of-flight mass spectrometer (AccuTOF-DART; JEOL USA, Inc., Peabody, MA, USA) equipped with a DART interface (IonSense Inc., Saugus, MA, USA). The gas heater was set to 200 °C, the glow discharge needle at 3.5 kV, and helium gas flow at 2.5 L/min. Under these conditions, mostly protonated ([M + H]^+^) molecules are observed, but protonation can occur with dehydration in alcohols to produce ([M + H − H_2_O]^+^) compounds.

The DART MS procedure was adapted from Yew *et al*.^33^. The androconia on forewings, hindwings, and control areas of CO_2_-anesthetized butterflies were rubbed with fine stainless steel forceps pre-cleaned with 70% ethanol and hexane. The tip of the forceps, often grasping a sample of scales and/or hairs, was held in the DART ion source. The sample was held in various orientations in the gas stream until peaks of chemical components started to appear. Five individuals of each condition were used for the measurements. Data processing including calibration, centroiding, spectral processing, and background subtraction was performed using Mass Center software (JEOL USA). All spectra were acquired in positive ion mode, and undiluted polyethylene glycol (Sigma-Aldrich, St. Louis, MO, USA) was used as an external calibrant. Elemental composition was predicted from exact mass measurements using Mass Mountaineer (V1.0.3.5; RBC Software, Portsmouth, NH, USA). The identity of the ion is assigned when the observed m/z of the protonated species is less than ±0.005 Da of the calculated value for the MSP.

### MSP extraction and Gas Chromatography-Mass Spectrometry (GCMS) analyses

The butterflies were anesthetized with CO_2_ and the wings cut with fine scissors at their base on the thorax. Both forewings and both hindwings were placed for 30 minutes in glass vials (Wheaton, Millville, NJ, USA) containing 500 μL of hexane with 10 μg.mL^−1^ of methyl stearate (Sigma-Aldrich) as an internal standard. Vials were pre-rinsed with hexane before the experiment, and the tools used to handle the butterflies or the wings were cleaned after treating each individual. MSP extractions were done between 2 and 3 pm. Collection time was held constant in order to avoid the confounding effects of daily fluctuations in pheromone titers. The extracts were stored at −20 °C.

A first set of analyses contained WS-27, DS-17, and DS-27 extracts (corresponding to results shown on Figs 3,4a,b and 5a) and was carried out on a GCMS QP2010 system (Shimadzu, Kyoto, Japan) equipped with a DB-5 column (5%-Phenyl-methylpolysiloxane column.30 m length, 0.32 i.d., 0.25 μm-film thickness, Agilent, Santa Clara, CA, USA). A second set of analyses compared WS-17 and DS-17 male MSP extracts (shown on Figs 4c,d and 5b) and was done on a Shimadzu Gas-Chromatography-QQQ Mass Spectrometer equipped with the same DB-5 column as described above. Ionization was achieved by electron ionization (EI) at 70 eV. One μL of each extract was injected splitless, with the injector temperature set up at 250 °C. Helium was used as carrier gas, with the flow set at 1.9 mL/min. The column temperature gradient began at 50 °C, increased to 210 °C at a rate of 35 °C/min, then increased to 280 °C at a rate of 3 °C/min. The detector was set to unit mass resolution and 3 scans/sec, from m/z 37 to 700. Chromatograms and mass spectra were analyzed using GCMSsolution software v.4.11 (Shimadzu). Relative amounts for each MSP were calculated by normalizing the area under each MSP peak to the area of the peak corresponding to the spiked standard.

Derivatization of double bonds by dimethyl disulfide (Sigma-Aldrich) was performed according to Buser *et al*.^61^ and the products analyzed by GCMS with the same parameters as described above.

### RNA extraction and Quantitative PCR (qPCR)

Eight days old male butterflies were placed in plastic containers, anesthetized with CO_2_, flash frozen in liquid nitrogen and stored at −80 °C until sample extraction. The androconial region was cut from their wings, on ice, under a stereomicroscope. The androconial scales were gently removed from the sample with a fine brush. The androconial tissue (the anterior and the posterior wing androconia from left and right wings) of 10 males was pooled in the same tube, and 3 biological and 3 technical replicates were done for each seasonal form.

RNA was extracted using TRIzol Reagent (Ambion, Austin, TX, USA) according to manufacturer’s instructions, and treated with TURBO DNA-free Kit (AM1907, Life Technologies, Carlsbad, CA, USA). Complementary DNA was synthesized using Superscript III Reverse transcriptase (18080-044, Life Technologies). Five ng of cDNA were used for qPCR with the KAPA SYBR FAST qPCR Kit (KK4604, KAPA Biosystems, Wilmington, MA, USA) and the experiment run on an Applied Biosystems 7900HT Fast Real-Time PCR system. The primers used are described in Supplementary Table 1.

Relative transcript levels were calculated using the N^−ΔΔCt^ method, where N is the primer efficiency calculated for each gene^62^. The Ct values were normalized to the reference gene *EF1α* and to one of the WS reference samples. Primer efficiencies were obtained by performing qPCR using 0.25, 2.5 or 5 ng of cDNA from WT butterflies with three technical replicates.

### Statistical analyses

Amounts of MSP were analyzed with a one-way multivariate analysis of variance (MANOVA), followed by univariate ANOVA to explore the effect on each MSP independently. A Bonferroni correction was applied to adjust for multiple pairwise comparisons. Two different GCMS instruments were used for the quantification of MSPs. However, because this factor could not be added as random factor in the MANOVA, data obtained from each instrument were analyzed separately. Hotelling-Lawley’s Trace, Pillai’s Trace, Roy’s Largest Root, and Wilk’s Lambda were all computed, but because the statistical outcome was similar for all of them only Pillai’s Trace was reported here. To ensure normality and homoscedasticity, MSP amounts were square-root transformed and ratios were inverse-transformed. Normality within group was checked with the Shapiro-Wilk test and homoscedasticity with Levene’s test. Multivariate normality was assessed using Mardia’s multivariate normality test and chi-square quantile-quantile plots. Multivariate outlier detection was done based on robust Mahalanobis distance. All analyses were carried out using the R language and environment for statistical graphics and computing^63^, and associated packages (*MVN* v.4.0^64^ and *car*^65^). Strength of shift in MSP amounts was calculated as standardized mean differences. We used Hedges’ g, which is a standardized effect size expressed in units of standard deviation^66^. R package *compute*.*es*^67^ was used to calculate Hedges’ g and its bootstrapped 95% confidence interval for the difference between DS-17 and DS-27, and between WS-27 and WS-17 (for effect of adult temperatures), and between WS-27 and DS-27, and WS-17 and DS-17 (for effect of developmental temperatures). For qPCR analysis, once agreement between technical replicates was obtained, differences in relative expression of pheromone biosynthetic genes were tested for significance with a Pair Wise Fixed Reallocation Randomization Test in the Relative Expression Software Tool (REST, 2009; http://rest.gene-quantification.info/)^68^. 5000 randomizations were done. DART, pheromone mass and ratio graphs were built with OriginPro 9.1.0 (OriginLab, Northampton, MA, USA, https://www.OriginLab.com).

## Additional Information

**How to cite this article**: Dion, E. *et al*. Phenotypic plasticity in sex pheromone production in *Bicyclus anynana* butterflies. *Sci. Rep.* **6**, 39002; doi: 10.1038/srep39002 (2016).

**Publisher's note:** Springer Nature remains neutral with regard to jurisdictional claims in published maps and institutional affiliations.

## Supplementary Material

Supplementary Information

## Figures and Tables

**Table 1:** MANOVA and univariate ANOVA analyses outcomes comparing amounts of MSPs between WS-27 and DS-17 males, WS-27 and DS-27 males, WS-17 and DS-17 males, and ratios of MSPs between DS-17 and WS-27 males.

		MSP1	MSP2	MSP3
Temperature treatment effects				
Forewings		Pillai’s Trace = 0.82 Approx. F = 8.79 *p* = *2*.*85e*^−*7*^		
Hindwings		Pillai’s Trace = 0.86 Approx. F = 9.01 *p* = *2*.*41e*^−*7*^		
WS-27 versus DS-17	*Forewings*	F_1,32_ = 31.76 *Adj*. *p* = *3*.*47e*^−*6*^	F_1,32_ = 4.99 Adj. p = 0.07	F_1,32_ = 52.70 *Adj*. *p* = *3*.*63e*^−*8*^
	*Hindwings*	F_1,32_ = 26.65 *Adj*. *p* = *3*.*24e*^−*5*^	F_1,32_ = 8.69 *Adj*. *p* = *1*.*06e*^−*2*^	F_1,32_ = 23.55 *p* = *3*.*81e*^−*5*^
WS-27versus DS-27	*Forewings*	F_1,32_ = 13.11 *Adj*. *p* = *1*.*04e*^−*4*^	Not detected	F_1,32_ = 8.52 *Adj*. *p* = *6*.*48e*^−*3*^
	*Hindwings*	F_1,32_ = 12.90 *Adj*. *p* = *2*.*40e*^−*3*^	F_1,32_ = 4.01 Adj. p = 0.11	F_1,32_ = 8.66 *Adj*. *p* = *6*.*33e*^−*3*^
**Temperature treatment effectForewingsHindwings**		**Pillai’s Trace = 0.32 Approx. F = 3.92 *p* = *0*.*04*Pillai’s Trace = 0.34 Approx. F = 2.37 p = 0.11**		
WS-17 versus DS-17	*Forewings*	F_1,18_ = 0.99 Adj. p = 0.33	Not detected	F_1,18_ = 4.74 *Adj*. *p* = *0*.*043*
	*Hindwings*	F_1,18_ = 2.70 Adj. p = 0.12	F_1,18_ = 0.41 Adj. p = 0.53	F_1,18_ = 7.63 *Adj*. *p* = *0*.*014*
		**MSP1/MSP3**	**MSP2/MSP1**	**MSP2/MSP3**
**Temperature treatment effect**		**Pillai’s Trace = 0.11 Approx. F = 1.13 p = 0.35**		
Ratios in WS-27versus ratios in DS-17	*Both wings*	F_1,32_ = 0.06 p = 0.81	F_1,32_ = 3.20 p = 0.083	F_1,32_ = 3.10 p = 0.09

P. adjust are adjusted p values after Bonferroni correction (BH, Holmes, Hochberg, Hommel and BY corrections were applied to data as well, but as they yielded similar p values, only the ones after Bonferroni correction were used), and p values in italics indicate significant differences between treatments.
