# Supplementary material for: Phenotypic plasticity in sex pheromone production in Bicyclus anynana butterflies
Source: Sci Rep. 2016 Dec 14;6:39002. doi: 10.1038/srep39002 (PMC5155268; doi:10.1038/srep39002)
Supplement: Supplementary Information [file srep39002-s1.pdf]

Phenotypic plasticity in sex pheromone production in *Bicyclus anynana* butterflies

Emilie Dion, Antónia Monteiro and Joanne Y. Yew

Supplementary materials

Supplementary Table 1. Primers used in this study.

Gene	Primers	GeneBank accession number
Elongation Factor-1 α (EF-1 α)	Forward: 5’- GTGGGCGTCAACAAAATGGA-3’<br>Reverse: 3’- GCAAAAACAACGAT-5’	KM923784.1
Fatty Acyl-CoA Reductase1 (Ban-wFAR1)	Forward: 5’-TCTCTTGGTGCGGGAGAAAC-3’<br>Reverse: 3’-AGCCAGGAGTTCCTCATCCT-5’	JQ978770.1
Fatty Acyl-CoA Reductase2 (Ban-wFAR2)	Forward: 5’-CGCTGCAACCACAAAGTTCA-3’<br>Reverse: 3’- GACACCGGTGCTGGATACAA-5’	JQ978771.1
Fatty Acyl Δ11- desaturase (Ban-Δ11)	Forward: 5’-ACCTCACCATCATGCGCTAC-3’<br>Reverse: 3’-GCTCCGCCGTTTTGTAATCC-5’	JQ978772.1

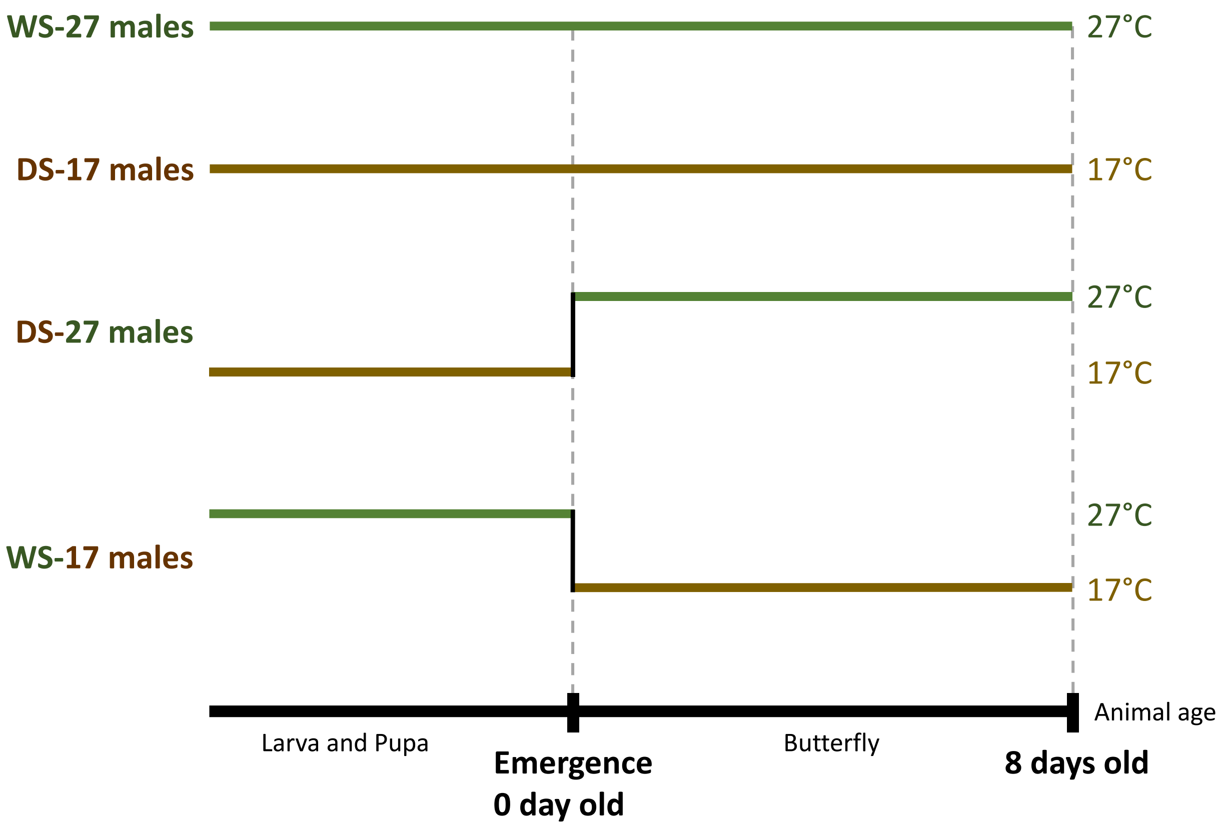

Supplementary Figure 1. Diagram of the different rearing temperature conditions used in the study. WS-27 and DS-17 males were kept their whole lives at 27°C and 17°C, respectively; DS-27 males were reared as larvae and pupae at 17°C and transferred to 27°C upon emergence, and WS-17 males were reared as larvae and pupae at 27°C then transferred to 17°C upon emergence.

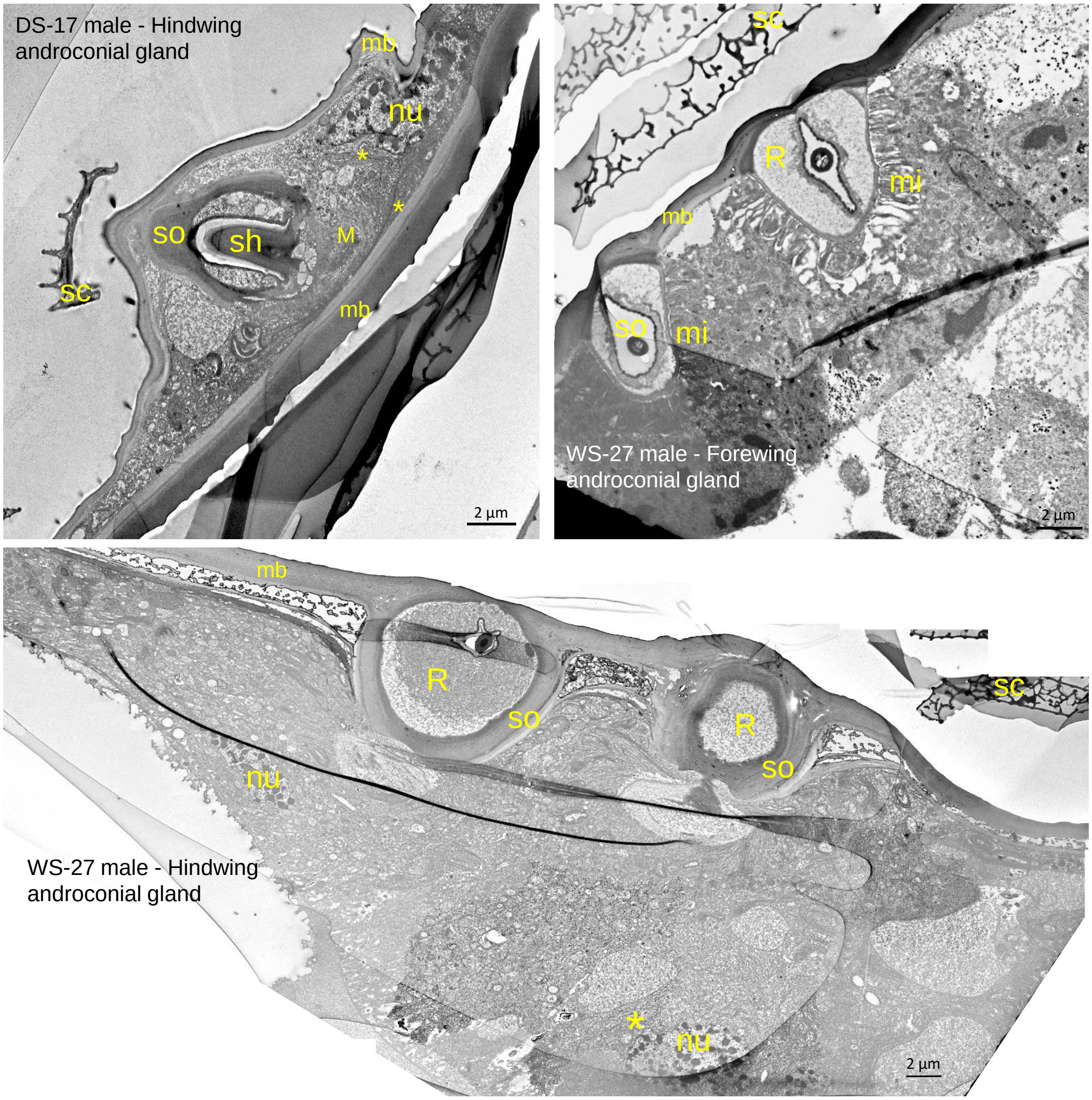

Supplementary Figure 2. TEM micrographs of a forewing and hindwing androconial gland of a WS-27 male, and of the androconial gland of a DS-17 male. Are shown: scales (sc), sockets (so), scale shafts (sh), wing membranes (mb), nucleus (nu), microvilli (mi), mitochondria (M), reservoir (R), tracheas (T), and endoplasmic reticulum (*).

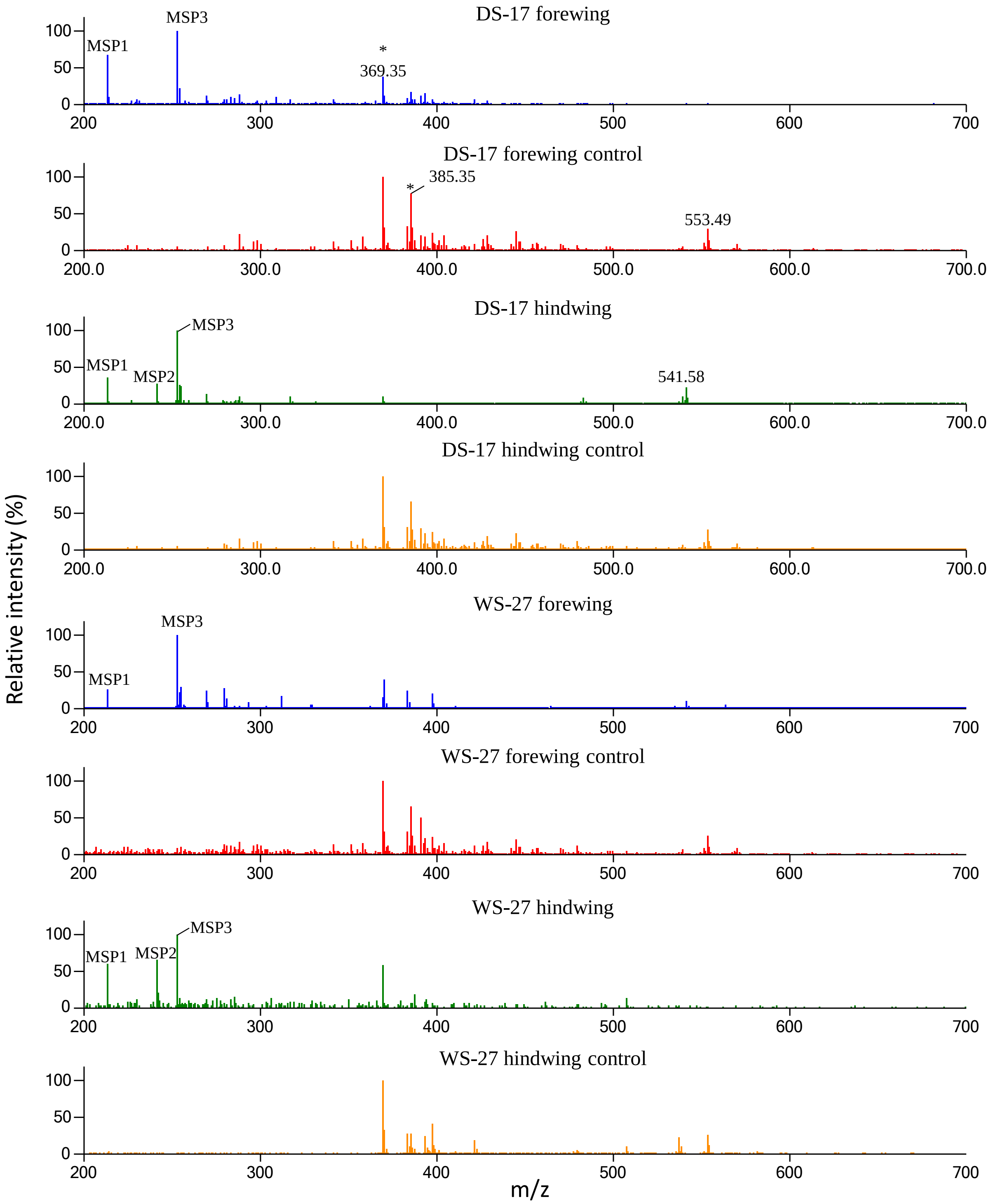

Supplementary Figure 3. Full range DART MS profiles of forewings and hindwings from 8 day old males. Common background contaminants derived from plasticizers found in containers, tools, and gloves are indicated by *. The signals detected at m/z 541.58 and 553.49 likely correspond to lipids from internal cellular stores.

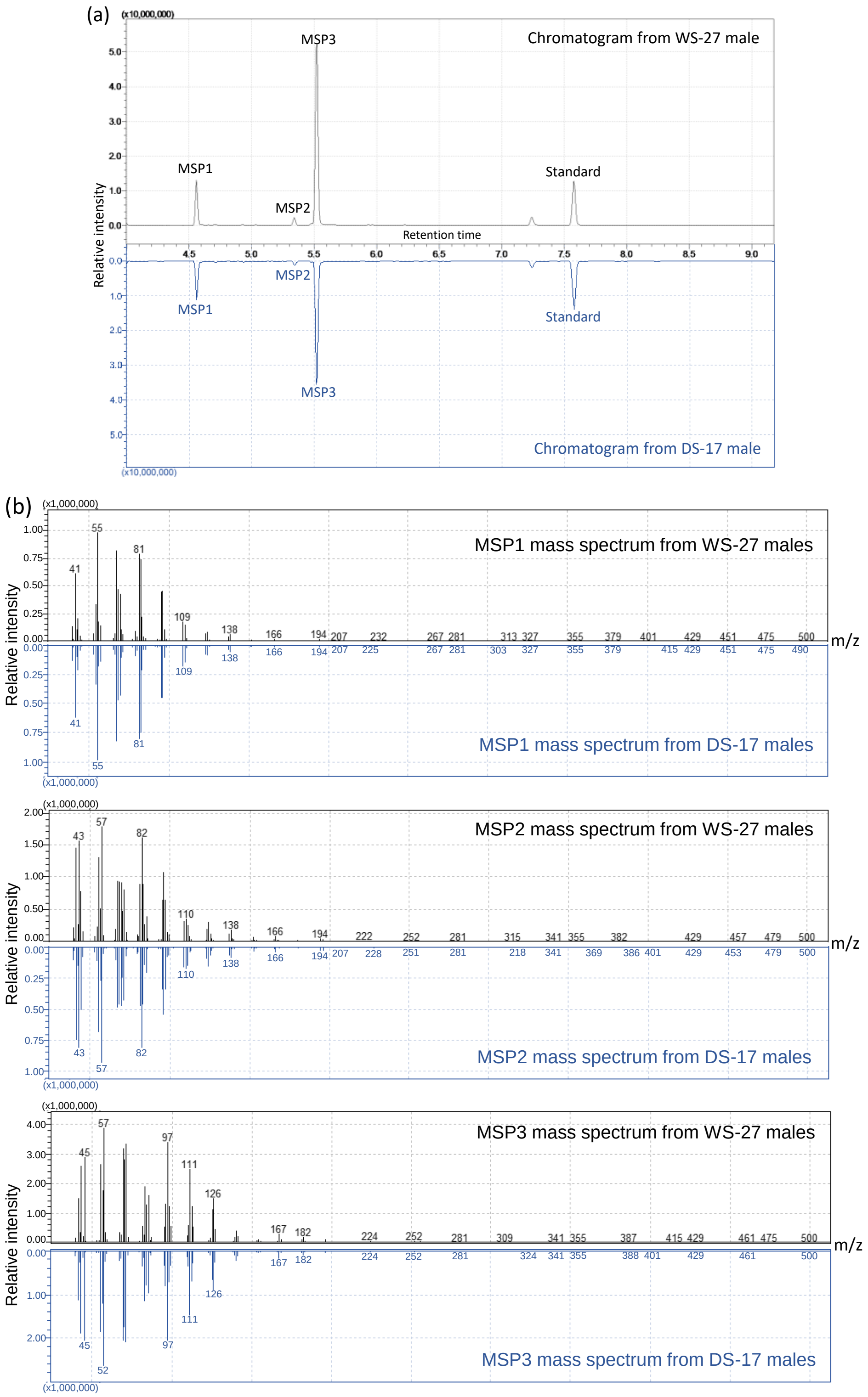

Supplementary Figure 4. GCMS analysis of MSP extracts from WS (in black) and DS (in blue) male wings show similar chromatogram profiles. (a) Retention times are the same for each MSP in both seasonal form wing extracts. (b) Electron ionization mass spectra for each MSP component are similar for both seasonal forms, confirming the same chemical structure.

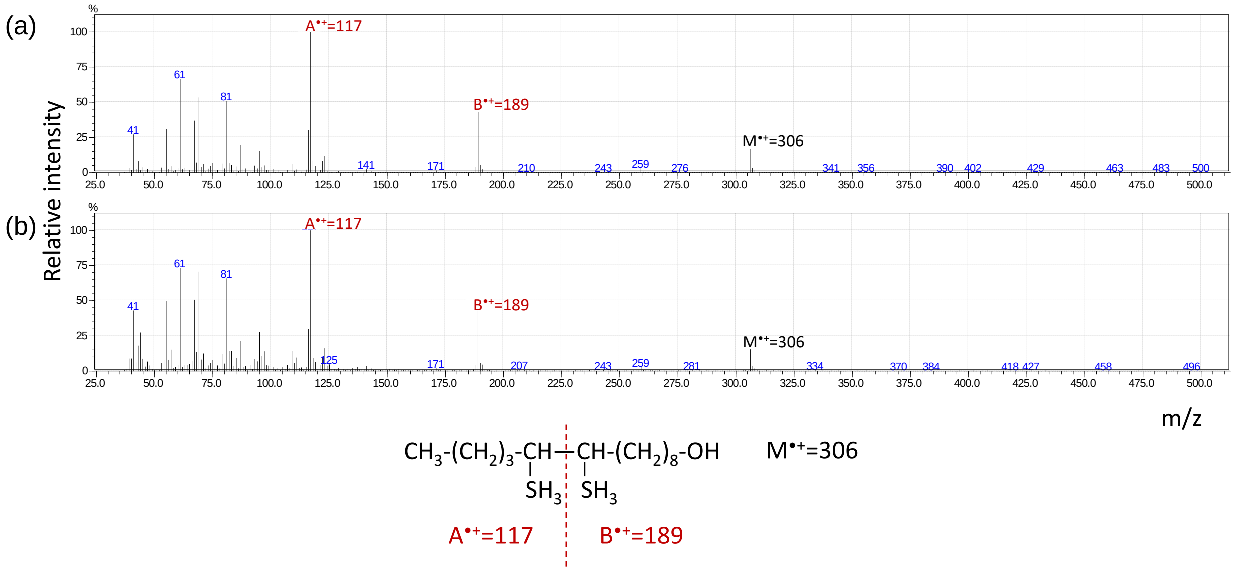

Supplementary Figure 5. The double bond is at the same position in MSP1 from WS-27 and from DS-17 males. Mass spectra of the dimethyl disulfide adducts of MSP1 from WS-27 (a) and DS-17 (b) males, after extraction from forewings and hindwings together. The 3 diagnostic mass peaks (with m/z indicated above each peak in red and black) correspond to the $A^{\bullet+}$ fragment (the aliphatic end of the molecule) and $B^{\bullet+}$ fragment (alcoholic end of the molecule). $M^{\bullet+}$ is the molecular ion. n(WS-27) and n(DS-17)=3 males.

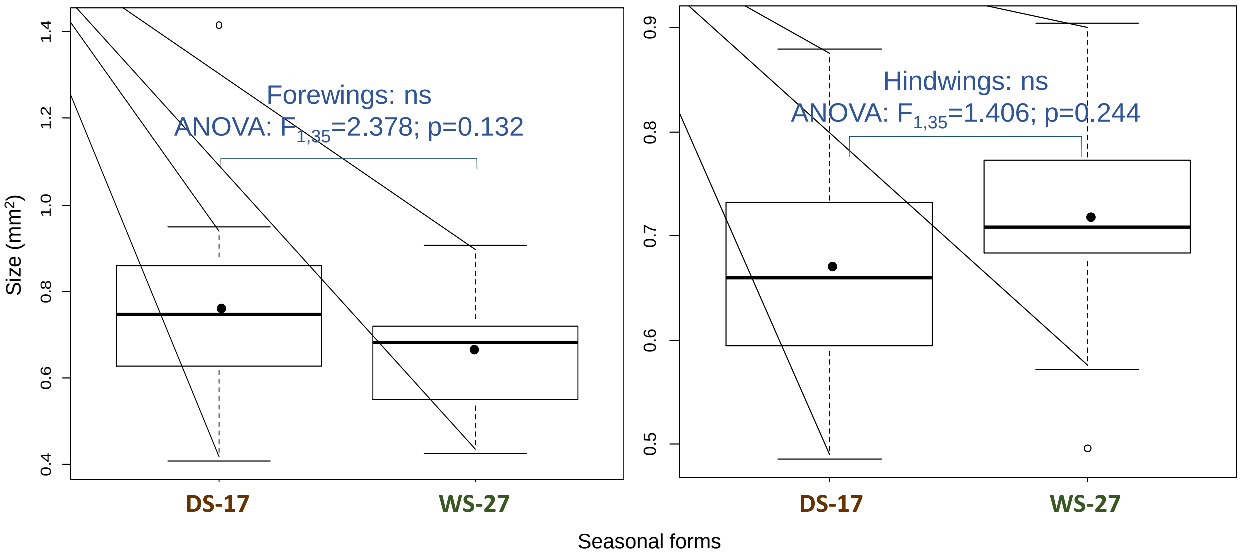

Supplementary Figure 6. DS-17 and WS-27 androconia have similar sizes on both wings. The horizontal line in each box is the median size of hindwing and forewing androconia, and the point in each box is the mean. The 25th and 75th percentiles are contained within the outline of the boxes, and the horizontal lines above and below each box show the 1.5 times inter-quartile range of the data. n(WS-27)=20 and n(DS-17)=23 wings from different males; ns = not significant. Androconia were measured using the method described in Nieberding et al. (2012) with the software ImageJ (Schneider et al., 2012; http://imagej.net/ImageJ).

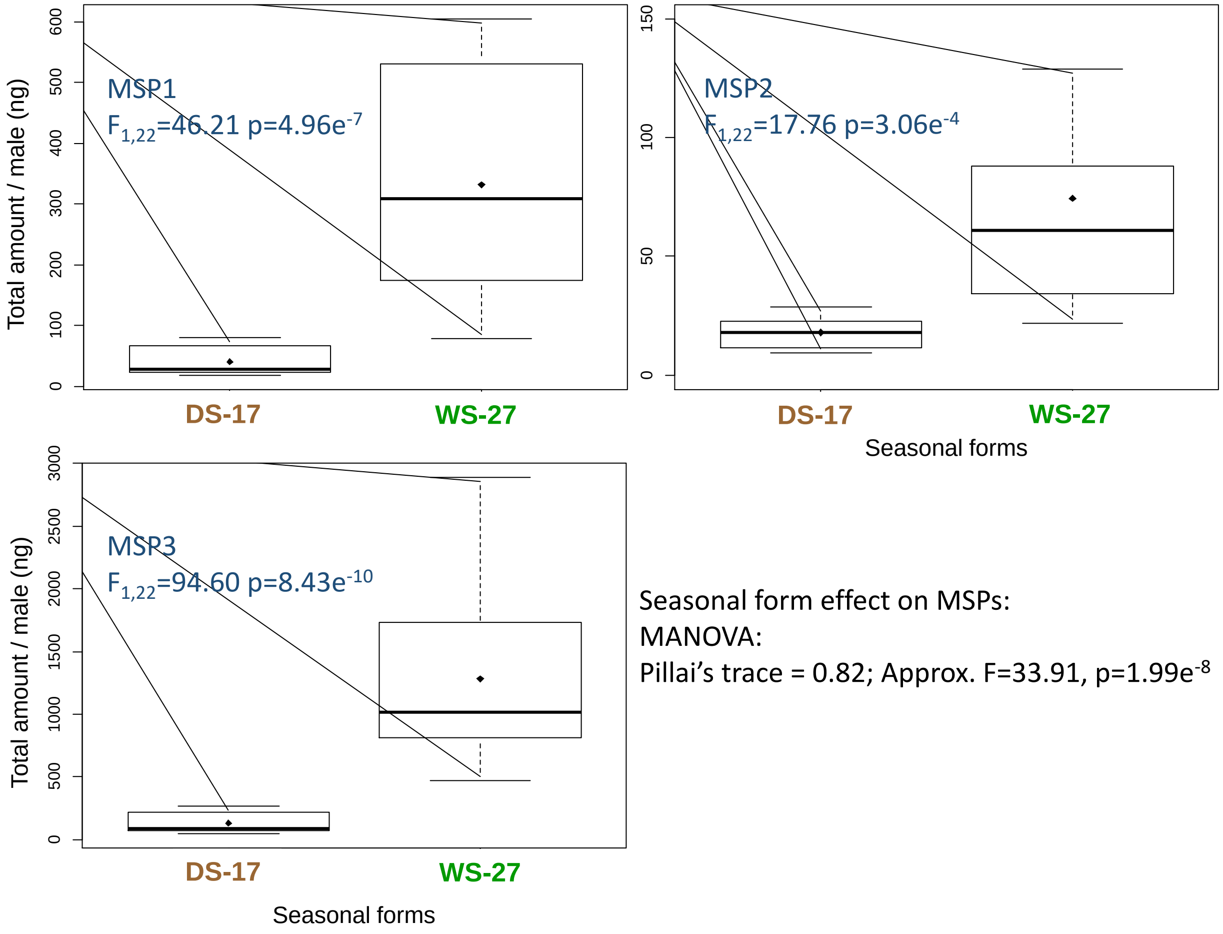

Supplementary Figure 7. DS-17 males already produce significantly less MSP than WS-27 males at 3 days old. The horizontal line in each box is the median amount of each MSP, and the point in each box is the mean. The 25 and 75 percentiles are contained within the outline of the boxes, and the horizontal lines above and below each box shows the 1.5 times inter-quartile range of the data. n(WS-27)=18 and n(DS-17)=6 males. MSP were extracted from both forewing and hindwing together and amounts were determined with GCMS. Amounts were analyzed with a one-way MANOVA (see details in the method section of the main document).

References:

Nieberding, C.M., Fischer, K., Saastamoinen, M., Allen, C.E., Wallin, E.A., Hedenstrom E. & Brakefield, P.M. Cracking the olfactory code of a butterfly: the scent of ageing. *Ecol. Lett.* **15**, 415-424 (2012).

Schneider, C.A., Rasband, W.S. & Eliceiri, K.W. NIH Image to ImageJ: 25 years of image analysis. *Nat. Meth.* **9**, 671-675 (2012).
